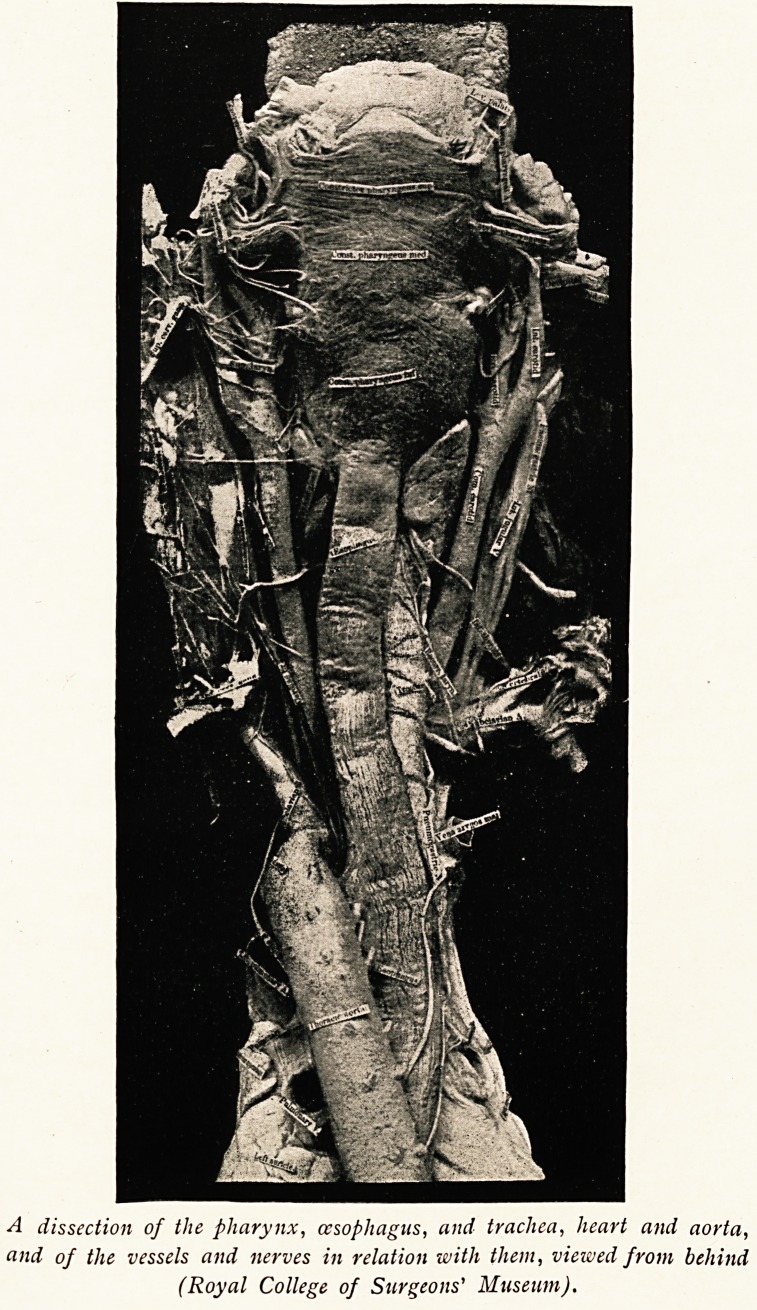# The Clinical Significance of Chronic Hoarseness and Loss of Voice

**Published:** 1901-09

**Authors:** P. Watson Williams

**Affiliations:** Physician in Charge of the Department for Diseases of the Throat, Bristol Royal Infirmary.


### THE CLINICAL SIGNIFICANCE OF CHRONIC HOARSENESS AND LOSS OF VOICE.

BY

P. Watson Williams, M.D. Lond.,

*Physician in Charge of the Department for Diseases of the Throat, Bristol Royal Infirmary.*

The clinical significance of such a commonplace symptom as hoarseness is very frequently under-estimated, firstly and chiefly, owing to the fact that hoarseness so commonly accompanies transient catarrhal affections of the throat and chest that neither doctor nor patient regards it as otherwise than a temporary inconvenience; and secondly, because when the graver causes of hoarseness have been recognised it becomes a very trivial complaint compared with the serious issues at stake.

Nevertheless, inasmuch as simple hoarseness is not seldom the only symptom noticed by the patient in the earlier stages of some grave organic affections, and as, furthermore, a laryngoscopic examination often furnishes the one and only piece of evidence enabling the early diagnosis of such organic disease to be made and successful treatment to be carried out, ere it is too late, *hoarseness which commences or persists without adequate and obvious cause should never fail to receive careful consideration, including a thorough inspection of the larynx.*

It would be superfluous to attempt to describe all the conditions that result in hoarseness, the purpose of this brief note being merely to emphasise the clinical significance of hoarseness and to direct attention to various classes of affections in which it is liable to arise.

### CATARRHAL AFFECTIONS.

Obviously inflammatory thickening due to catarrhal affections and growth or ulceration preventing due approximation of the vocal cords or causing loss of substance along the vocal cord margins, will result in hoarseness or complete loss of voice; but in most cases other symptoms will not only be present, but will have attracted the notice of the patient.

Hoarseness from chronic catarrh may be due to constipation, dyspepsia and various hepatic derangements, while gout as a possible cause of subacute or chronic laryngitis should always be borne in mind.

### CHRONIC INFECTIVE DISEASES.

In an adult persistent hoarseness with catarrhal inflammation of the larynx, especially if unilateral, should lead to the suspicion of tuberculosis, syphilis, or malignant disease. Quite recently a young lady was referred to the writer on account of weakness in the singing voice. She was able to speak without trouble, and had a fair speaking voice, which, however, became husky with much talking; but she was a singer, and the marked impairment in the singing voice, both as regards power and range, had caused her to seek advice. Examination of the larynx revealed nothing except a slight anæmia of the mucous membrane and somewhat weak movements of the vocal cords. Now inquiry into her general state of health elicited the history of a constant succession of slight colds for six months, and examination of the chest revealed defective breath sounds and harsh breathing at the apex of the right lung. Her pulse rate was 92, and she had a very strong family history of tuberculosis. The hoarseness and weakness of voice were probably due to muscular weakness and not to any laryngeal disease.

Apart from the general manifestations suggestive of tuberculous disease, a pronounced and persistently anæmic condition of the mucous membrane of the fauces and larynx is highly suspicious and not infrequently precedes the grosser signs of local tuberculous disease.

The majority of educated patients who are known to have pulmonary tuberculosis are aware that hoarseness is a serious symptom, and every medical practitioner realises the possibility of laryngeal complications, but it is certainly less generally recognised that hoarseness may be the precursor of the more tangible evidences of phthisis.

Of early syphilitic disease it is unnecessary to say anything beyond mentioning it as a cause of hoarseness which may be difficult, apart from other manifestations and the history of the patient, to differentiate from early phthisis and malignant disease.

### MALIGNANT GROWTHS.

A malignant growth of the larynx may first declare its presence by the occurrence of simple hoarseness. So deeply rooted in the minds of the public, and indeed of some practitioners, is the idea that cancer is always associated with pain, that a malignant growth as the possible cause of simple persistent hoarseness is unfortunately completely overlooked in a large proportion of cases, and thus a cancerous growth, which could be removed with excellent prospects of permanent eradication, may be, and often is, permitted to remain unnoticed till, with the later occurrence of pain and perhaps secondary glandular infiltration, suspicions are aroused too late for successful surgical interference. I have elsewhere[1] reproduced a drawing of a case of early epithelioma of one

[1] *Diseases of the Upper Respiratory Tract.* 4th Ed. Watson Williams. Plate xx., fig. 1.

vocal cord associated with hoarseness, and which was successfully removed by Dr. Middlemass Hunt. It is the earliest and most limited case of the kind, proved to be epithelioma by histological examination, that I have seen illustrated.

### PARALYTIC AFFECTIONS.

Paralysis or paresis of the vocal cords resulting in hoarseness may be due to so many causes that it is well to group them under three headings :

1. Inflammatory or other conditions causing more or less complete ankylosis of the crico-arytenoid joint.
2. Peripheral paralysis of the motor nerves of the laryngeal muscles.
3. Affections of the central nervous system.

(1) Ankylosis of the crico-arytenoid joint is frequently attended with hoarseness, even if unilateral, because the vocal cord is generally in the cadaveric position, and only rarely is the inflammatory exudation or other determining cause of ankylosis so completely absorbed as not in any way to interfere with the movements of the opposite cord. But these are local affections of the larynx and have but little bearing on general diseases.

(2) Peripheral paralysis of the recurrent laryngeal nerve fibres, either before or after leaving the vagus nerve, is one of the commonest causes of hoarseness, and often affords the one and only symptom leading to an early diagnosis of intra-thoracic aneurysm or other grave organic diseases.

It will suffice to classify the less rare causes of peripheral recurrent laryngeal paralysis as follows:—

*a.* Intracranial.—Meningeal thickening, syphilitic pachymeningitis, growths involving the accessory vagus nerve at the lower part of the medulla.

*b.* In the neck.—Any growths at the base of the skull involving the vagus nerves, enlarged glands, goitre or other enlargement of the thyroid gland, cancer of the œsophagus.

*A dissection of the pharynx, œsophagus, and trachea, heart and aorta, and of the vessels and nerves in relation with them, viewed from behind (Royal College of Surgeons' Museum).*

*c*. Intrathoracic.—Aneurysm of the aorta, or of the right innominate artery, tuberculous deposits at the apex of the right lung, mediastinal tumours, growths in the neighbourhood of the root of the left lung, and finally peripheral neuritis, as in multiple peripheral neuritis or that due to lead poisoning, rheumatism or other toxic matters in the blood.

The course followed by the recurrent nerve fibres through the neck and chest, and particularly the different anatomical arrangement on the two sides, will be seen from the accompanying plate. The "left vagus is seen passing down in front of the arch of the aorta, and here its recurrent branch passes beneath the aortic arch to ascend between the œsophagus and trachea. Obviously an aneurysm of the arch may cause pressure on either the left vagus or the left recurrent nerve, or on both, according to the situation and size of the aneurysm. Again, on the right side, the vagus nerve may be seen descending in front of the right subclavian artery, and the recurrent nerve hooking round this vessel to ascend behind the innominate artery so as to reach the sulcus between the œsophagus and trachea here. Plainly an aneurysm of the innominate or subclavian arteries may cause pressure on either the right vagus or recurrent laryngeal nerve. But an aneurysm of the aortic arch involving that portion which gives off the innominate artery may reach sufficiently high up to cause compression of both the right and left vagus nerves. . . . These remarks apply likewise to other intra-thoracic tumours involving these nerves."[1]

(3) Inasmuch as, in accordance with Semon's law, all progressive organic lesions resulting in paralysis of the vocal cords, the abductors are more prone to succumb than the adductors, chronic diseases of the central nervous system usually declare their presence by general symptoms and signs before the adductors are implicated and thus before hoarseness arises, for abductor paralysis does not cause hoarseness or loss of voice. Hence, although hoarseness or aphonia may supervene in cases of tabes dorsalis, bulbar paralysis, general

[1] *Ibid.* p. 265.

paralysis of the insane, etc., the hoarseness generally comes too late to be of any diagnostic import, while the so-called functional or hysterical aphonia is so widely recognised as to require no comment.

The long, but by no means exhaustive, list of organic diseases cited as being prone to be associated early in their development with hoarseness sufficiently indicates the importance of paying due attention to such a commonplace symptom if one is to avoid the mortification of realising too late that it was a constant danger signal, careful investigation of which might have warned us of the true nature of some latent disease.

## Figures and Tables

**Figure f1:**